# Modulating Mistranslation Potential of tRNA^Ser^ in *Saccharomyces cerevisiae*

**DOI:** 10.1534/genetics.119.302525

**Published:** 2019-09-04

**Authors:** Matthew D. Berg, Yanrui Zhu, Julie Genereaux, Bianca Y. Ruiz, Ricard A. Rodriguez-Mias, Tyler Allan, Alexander Bahcheli, Judit Villén, Christopher J. Brandl

**Affiliations:** * Department of Biochemistry, The University of Western Ontario, London, Ontario N6A 5C1, Canada; † Department of Genome Sciences, University of Washington, Seattle, Washington 98195

**Keywords:** serine tRNA, tRNA modifications, mistranslation, synthetic biology, statistical proteins

## Abstract

Transfer RNAs (tRNAs) read the genetic code, translating nucleic acid sequence into protein. For tRNA^Ser^ the anticodon does not specify its aminoacylation. For this reason, mutations in the tRNA^Ser^ anticodon can result in amino acid substitutions, a process called mistranslation. Previously, we found that tRNA^Ser^ with a proline anticodon was lethal to cells. However, by incorporating secondary mutations into the tRNA, mistranslation was dampened to a nonlethal level. The goal of this work was to identify second-site substitutions in tRNA^Ser^ that modulate mistranslation to different levels. Targeted changes to putative identity elements led to total loss of tRNA function or significantly impaired cell growth. However, through genetic selection, we identified 22 substitutions that allow nontoxic mistranslation. These secondary mutations are primarily in single-stranded regions or substitute G:U base pairs for Watson–Crick pairs. Many of the variants are more toxic at low temperature and upon impairing the rapid tRNA decay pathway. We suggest that the majority of the secondary mutations affect the stability of the tRNA in cells. The temperature sensitivity of the tRNAs allows conditional mistranslation. Proteomic analysis demonstrated that tRNA^Ser^ variants mistranslate to different extents with diminished growth correlating with increased mistranslation. When combined with a secondary mutation, other anticodon substitutions allow serine mistranslation at additional nonserine codons. These mistranslating tRNAs have applications in synthetic biology, by creating “statistical proteins,” which may display a wider range of activities or substrate specificities than the homogenous form.

## Abstract (precis)

Mistranslation, incorporating an amino acid not specified by the “standard” genetic code, has applications in research and synthetic biology. Since mistranslation is toxic, its level must be modulated. Using a serine tRNA with a proline anticodon, we identify...

MISTRANSLATION occurs when an amino acid that differs from that specified by the “standard” genetic code is incorporated into nascent proteins during translation. Although considered less frequently than protein modification, mistranslation plays a significant role in generating protein diversity. Mistranslation naturally occurs at a frequency of ∼1 in every 10^4^–10^5^ codons (Kramer and Farabaugh 2007; Drummond and Wilke 2009), with some conditions increasing this frequency (Santos *et al.* 1999; Bacher *et al.* 2007; Javid *et al.* 2014). Woese (1965) predicted that mistranslation was substantially greater during the evolution of the translational machinery, creating diversity that would allow proteins to probe phase-space. Mistranslation is also used in several systems as an adaptive response (Ling and Söll 2010; Moghal *et al.* 2014; Wu *et al.* 2014; Wang and Pan 2016). For example, in response to oxidative stress, *Escherichia coli*, yeast, and mammalian cells mistranslate methionine into proteins to sequester reactive oxygen species and protect cells from oxidative damage (Netzer *et al.* 2009; Wiltrout *et al.* 2012; Lee *et al.* 2014; Gomes *et al.* 2016; Schwartz and Pan 2017). In the archaeon *Aeropyrum pernix*, transfer RNA (tRNA)^Leu^ is misaminoacylated by methionyl-tRNA synthetase at low temperatures, which enhances enzyme activity (Schwartz and Pan 2016). *Mycoplasma* species use editing-defective synthetases to generate diversity and escape the host defense systems (Li *et al.* 2011). In other cases, mistranslation results in nearly complete codon reassignment. Yeasts of the *Candida* genus naturally evolved a tRNA^Ser^ variant that ambiguously decodes the leucine CUG codon mainly as serine (Massey *et al.* 2003; Paredes *et al.* 2012).

The first specificity step of translation is aminoacylation of a tRNA by its corresponding aminoacyl-tRNA synthetase [aaRS; reviewed in Pang *et al.* (2014)]. Each aaRS recognizes its cognate tRNAs from a pool of tRNAs with similar structure using structural elements and nucleotides within the tRNA called identity elements (Rich and RajBhandary 1976; de Duve 1988; Giegé *et al.* 1998). For many tRNA-aaRS interactions, the specificity is determined in large part by the anticodon. In yeast the exceptions to this are tRNA^Ser^ and tRNA^Ala^ (Giegé *et al.* 1998). The major identity elements for tRNA^Ser^ and tRNA^Ala^ are the variable arm, positioned 3′ to the anticodon stem, and a G3:U70 base pair, respectively. Because of the latter, inserting a G3:U70 base pair into other tRNAs results in misaminoacylation with alanine (McClain and Foss 1988; Francklyn and Schimmel 1989; Hoffman *et al.* 2017; Lant *et al.* 2017). In the case of tRNA^Ser^, changes to the anticodon misincorporate serine since the ribosome does not monitor the amino acid on the incoming tRNA (Chapeville *et al.* 1962). Post-transfer editing mechanisms exist to help maintain translation fidelity after aminoacylation. These involve editing domains that are part of the aaRS and free-standing proteins [reviewed in Ling *et al.* (2009)].

Mistranslation has applications in synthetic biology. tRNAs that misincorporate amino acids expand the diversity of expressed proteins, resulting in what Woese described as “statistical proteins” [Woese 1965; reviewed in Schimmel (2011)]. Statistical proteins have the potential to display a wider range of activities or substrate specificities than the homogeneous form. For example, generating antibodies that are heterogeneous mixtures, with each molecule containing one or two amino acid variants, could expand antigen recognition and be valuable for rapidly evolving antigens. Although tolerated and sometimes beneficial to cells, too much mistranslation can be lethal (Berg *et al.* 2017). Therefore, for mistranslation to have biological applications the activity of the mistranslating tRNA must be tuned so that it is below a toxicity threshold. Zimmerman *et al.* (2018) exploited the rapid tRNA decay (RTD) pathway to control tRNA levels in yeast. In this approach, mistranslating tRNAs are mutated to become substrates of the RTD pathway by destabilizing the acceptor stem (Whipple *et al.* 2011). The RTD pathway is controlled using an inducible *MET22* gene, where repression of *MET22* inhibits the RTD pathway and induces mistranslation by the mutant tRNA. Although effective, this approach also influences the levels of endogenous tRNAs.

Regulating other steps along the tRNA pathway allows the levels and/or activity of a tRNA to be controlled. These include steps during biosynthesis (*e.g.*, transcription, processing, 3′ CCA addition, and splicing), nuclear export, aminoacylation, and interaction with the translational machinery (Nissen *et al.* 1996; Dreher *et al.* 1999; Phizicky and Hopper 2010; Hopper 2013). tRNAs are also extensively modified in both the nucleus and cytoplasm (Jackman and Alfonzo 2013). The modified bases are identity elements for aaRS enzymes (Giegé *et al.* 1998), regulate codon-anticodon pairing (Gustilo *et al.* 2008; Wei *et al.* 2011), maintain the reading frame during decoding (Urbonavičius *et al.* 2001), and regulate the tRNA structure (Lorenz *et al.* 2017). The final aspect of tRNA regulation is their degradation through either the RTD pathway mentioned above, which degrades hypomodified and unstable tRNAs (Chernyakov *et al.* 2008; Whipple *et al.* 2011), or the nuclear exosome, which monitors tRNA modifications and 3′ end maturation (Kadaba *et al.* 2004; Schneider *et al.* 2007; Schmid and Jensen 2008).

Because of their toxicity (Berg *et al.* 2017), the applications of mistranslating tRNAs to research and biotechnology requires that their activity be regulated. The goal of this work was to identify a range of base changes in tRNA^Ser^ that would dampen tRNA function and fine-tune the extent of mistranslation for use in different applications and with a number of anticodon substitutions. Using a genetic suppression system that requires a proline codon be mistranslated as serine, we selected mistranslating tRNA^Ser^_UGG_ variants with a range of activities after random mutagenesis. Many of these had increased toxicity at low temperature and upon inhibiting the RTD pathway, suggesting that they destabilize the tRNA, and enabling temperature-sensitive induction of mistranslation. Through targeted changes to predicted identity elements, we also identified substitutions in the acceptor stem and discriminator base that diminish lethality and allow mistranslation. Proteomic analysis demonstrated that tRNA^Ser^ variants mistranslate to different extents with diminished growth correlating with increased mistranslation. Thus, by altering nucleotides in tRNA^Ser^ it is possible to decrease tRNA^Ser^ function and mistranslate with various efficiencies. In addition, we demonstrate that in combination with the correct secondary mutation, the anticodon of the tRNA^Ser^ can be mutated to mistranslate arginine, glutamine, phenylalanine, and ochre stop codons, expanding the mistranslation potential of this tRNA.

## Materials and Methods

### Yeast strains and growth

All yeast strains are derivatives of the wild-type haploid strains BY4741 and BY4742 (Supplemental Material, Table S1; Winzeler and Davis 1997). The *tti2* disruption strains covered by either *TTI2* (CY6963), *tti2**-L187P (*CY7020), or *tti2**-Q276TAA* (CY6874) on a centromeric plasmid have been described (Hoffman *et al.* 2016). The *tti2* disruption strain with *tti2**-L187R* and tRNA^Ser^_UCU_-G26A was made by transforming *tti2**-L187R* on an *LEU2* centromeric plasmid into CY6963 along with *sup17**(UCU)-G26A* on an *HIS3* centromeric plasmid. The wild-type *TTI2* on a *URA3* plasmid was lost by counter selection on 5-fluoroorotic acid to generate CY8150. The *met22*Δ strain (CY8588) and its isogenic *MET22* control (CY8589) were derived from a spore colony of the magic marker strain in the BY4743 diploid background (Tong *et al.* 2001).

Yeast strains were grown in yeast peptone media containing 2% glucose or synthetic media supplemented with nitrogenous bases and amino acids at 30° unless otherwise indicated. For spot assays on plates, strains were grown to saturation in selective medium, OD_600_ was normalized, and cultures were spotted in 10-fold serial dilutions. To quantitate growth on solid media, cells were plated after dilution to obtain single colonies. Colony size was measured using ImageJ (v1.52h; Schneider *et al.* 2012). Growth curves were generated by diluting saturated cultures to OD_600_ ∼0.1 in minimal media and incubating at 30°. OD_600_ was measured every 15 min using a BioTek Epoch 2 microplate spectrophotometer for 24 hr. Doubling time was calculated using the R package “growthcurver” (Sprouffske and Wagner 2016).

### Plasmid constructs

*SUP17* (pCB3076) and *sup17**(UGG)* (pCB3082) expressed in YCplac33 have been previously described (Berg *et al.* 2017). Derivatives of YCplac33-*sup17**(UGG)* with mutations at G9A (pCB4020), A20bG (pCB4021), G26A (pCB4023), and C40T (pCB4022) were obtained previously (Berg *et al.* 2017), whereas A4G (pCB4097), C5T (pCB4080), C12T (pCB4102), G15A (pCB4106), G18A (pCB4114), T20C (pCB4088), A29G (pCB4087), T33G (pCB4084), A38C (pCB4081), T39G (pCB4093), T44G (pCB4074), G45A (pCB4075), Ge23A (pCB4090), Ce21T (pCB4072), C48T (pCB4079), T51C (pCB4101), A59G (pCB4098), and T60G (pCB4089) were obtained through genetic selection.

*sup17**(UGG)-VA*Δ was engineered using two-step mutagenic PCR with primers UG5953/VI1383 and UG5954/VI1382 (oligonucleotides are listed in Table S2) in the first round, with pCB3082 as template. Products from the first reaction were amplified with outside primers UG5953/UG5954 and cloned into YCplac33 as an *Eco*RI fragment after first subcloning into pGEM-Teasy (Promega, Madison, WI) to give pCB4177. *sup17**(UGG)-G11:C24* was cloned using the same strategy and inside primers VJ2697/VJ2698 to give pCB4191. *sup17**(UGG)-VA*Δ*1*, *sup17**(UGG)-VA-G:C*, *sup17**(UGG)-G73A*, and *sup17**(UGG)-G73C* were similarly engineered with inside primers VK4593/VK4594, VJ2766/VJ2767, WA5536/WA5537, or WA6571/WA6572, and cloned into YCplac33 *Hin*dIII/*Eco*RI to give pCB4234, pCB4206, pCB4259, and pCB4275, respectively.

Acceptor stem variants with flipped bases at positions 1:72 and 2:71 were synthesized by Life Technologies and cloned into YCplac33 as *Eco*RI/*Pst*I fragments to give pCB4268 and pCB4274, respectively.

The variant with mutations at positions 3:70 was made sequentially. First, the G70C mutation was made by two-step PCR using outside primer UG5953/UG5954, inside primers VL5002/VL5003, and template pCB3082. The final product of that PCR was used as template for another two-step PCR using the same outside primers and inside primers VF8661/VF8662 to make the C3G mutation. The product was cloned into YCplac33 as an *Eco*RI fragment to give pCB4254. The variant with mutations at positions 4:69 was similarly made. First, the A4T mutation was made by two-step PCR using inside primers WF1163/WF1164 and template pCB3082. The final product was used as template to introduce the T69A mutation using inside primers WF1165/WF1166. The product was cloned into YCplac33 as an *Hin*dIII/*Eco*RI fragment to give pCB4333.

*sup17**(UCU)* and *sup17**(UCU)-G9A* were made by two-step mutagenic PCR with outside primers UG5953/UG5954 and inside primers VJ2409/VJ2410 from template pCB3082 or pCB4020, respectively. The products were cloned as *Eco*RI fragments into pRS303 (Sikorski and Hieter 1989) after first subcloning into pGEM-Teasy (Promega), giving pCB4215 or pCB4216, respectively. *sup17**(UCU)-G9A* was also cloned into YCplac33 to give pCB4120. *sup17**(UCU)-G26A* was similarly made using template pCB3082, and inside primers VL4943/VL4945 giving pCB4257. The following derivatives were made by two-step PCR using outside primers UG5953 and UG5954. Final products were cloned into YCplac33 as an *Eco*RI fragment after first subcloning into pGEM-Teasy (Promega). Template, inside primers, and construct are in parentheses: *sup17**(UUA)* (pCB3082, VK4595/VK4596, pCB4235), *sup17**(UUA)-G9A* (pCB4020, VK4595/VK4596, pCB4236), *sup17**(CUG)-G26A* (pCB4023, VF7765/VF7766, pCB4311), and *sup17**(GAA)-G26A* (pCB3082, WC8504/WC8505, pCB4358).

*TTI2*, *tti2**-L187P*, and *tti2**-Q276TAA* constructs have been described (Hoffman *et al.* 2016). The *tti2**-L187R* construct was made by two-step PCR using outside primers 5693-1/5693-2, inside primers 6856-1/6856-2, and template pCB2134. The product was cloned into the wild-type *DED1pr-TTI2* vector as a *Not*I-*Sac*I fragment to give pCB2865.

The centromeric plasmid containing *HSE-eGFP* was kindly provided by Onn Brandman (Stanford University) (Brandman *et al.* 2012). The *URA3* marker on the plasmid was switched to *HIS3* using pUH7 (Cross 1997).

### Selection of variants of sup17(UGG) that suppress tti2-L187P

Selection of mutant *sup17**(UGG)* alleles that support viability and suppress *tti2**-L187P* was performed as previously described (Berg *et al.* 2017). Briefly, YCplac33-*sup17**(UGG)* were UV-irradiated and transformed into CY7020. Ura+ transformants were screened for growth on YPD containing 5% ethanol. The YCplac33 plasmids were then isolated, sequenced, and transformed back into CY7020 to analyze growth.

### Fluorescence heat shock reporter

Yeast strains containing the heat shock response element (*HSE)-eGFP* reporter and a YCplac33-*sup17**(UGG)* allele were grown to stationary phase in medium lacking histidine and uracil, diluted 1:20 in the same medium and grown for 6 hr at 30°. Cell densities were normalized to OD_600_ before measuring fluorescence. Fluorescence was measured with a BioTek Synergy H1 microplate reader at an emission wavelength of 528 nm using Gen5 2.08 software.

### Mistranslation quantification using mass spectrometry

Starter cultures of yeast strains containing YCplac33-*sup17**(UGG)* variants were grown to stationary phase in medium lacking uracil, diluted 1:20 in 10 ml of the same medium and grown for 19 hr at 30°. Cell pellets from the resulting 10 ml of yeast culture were resuspended in a denaturing lysis buffer (8 M urea, 50 mM Tris, pH 8.2, 75 mM NaCl). Cells were lysed by bead-beating with 0.5 mm glass beads at 4°. Lysates were cleared by centrifugation at 21,000 × *g* for 10 min at 4° and protein concentration was determined by BCA assay (Pierce, Thermo Fisher Scientific). Proteins were reduced with 5 mM dithiothreitol for 30 min at 55°, alkylated with 15 mM iodoacetamide for 30 min at room temperature, and the alkylation was quenched with additional 5 mM dithiothreitol for 15 min at room temperature. For each sample, 100 μg of protein was diluted 1:2 with 50 mM Tris, pH 8.9, and digested overnight at room temperature with 1 μg LysC (Wako Chemicals). Digestions were acidified to pH 2 with trifluoroacetic acid and desalted over Empore C18 stage tips (Rappsilber *et al.* 2007).

Peptide samples were resuspended in 4% acetonitrile, 3% formic acid, and subjected to liquid chromatography coupled to tandem mass spectrometry. Samples were loaded onto a fused silica capillary column packed with 1.9 μm Reprosil-Pur C18 AQ reversed-phase resin and separated using a gradient of 8–30% acetonitrile in 0.125% formic acid delivered at 250 nl/min over 95 min, with a total 120-min acquisition time. Peptides were analyzed online on the linear ion trap Orbitrap (LTQ Velos Orbitrap; Thermo Fisher Scientific) hybrid mass spectrometer using a data-dependent acquisition method. For each cycle, one full mass spectrometry scan was acquired from 350 to 1500 m/z at 60,000 resolution on the Orbitrap, with fill target of 3E6 ions and maximum injection time of 500 msec, followed by up to 20 tandem mass spectrometry on the 20 most-intense precursor ions fragmented by collision-induced dissociation and acquired in the ion trap with a 3E4 fill target and 100 msec of maximum injection time.

Raw files were converted to the open mzXML format and searched against the *Saccharomyces* Genome Database yeast protein sequence database (downloaded in 2014) using Comet (release 2015.01; Eng *et al.* 2013). The false discovery rate (FDR) was estimated using a target-decoy strategy (Elias and Gygi 2007). Data were filtered to 1% FDR at the peptide-spectrum match level using Percolator (2017; Käll *et al.* 2007). To identify peptides with serine substitutions at proline sites, the search was conducted with a variable modification corresponding to the mass shift of a proline to serine substitution at proline positions. A maximum of two proline to serine substitutions per peptide were allowed, as the low rate of substitution we observed was considered unlikely to produce detectable peptides with multiple substitutions. Additional search parameters were cleavage C-terminal to lysine with a maximum of two missed cleavages, constant modification of carbamidomethylation on cysteines, variable modifications of methionine oxidation and N-terminal protein acetylation, tolerance of 50 ppm for precursor masses, and 0.36 kDa with 0.11 offset for fragment ions.

To estimate the frequency of substitution, we calculated the fraction of unique peptides containing the proline sites for which the serine-substituted version of the peptide was also detected. To minimize the FDR among mistranslated peptides, we applied additional, more stringent filtering. Serine-substituted peptides with additional modifications (methionine oxidation or N-terminal acetylation) or those in which the corresponding wild-type peptide was not present in the data set were filtered out. For codon specific substitution analysis, an additional filtering step was applied. Only peptides with a single proline instance were considered so as to rule out potential site localization issues.

### Data availability

Strains and plasmids are available upon request. The authors affirm that all data necessary for confirming the conclusions of the article are present within the article, figures, and tables. File S1 contains all supplemental figures and tables. Supplemental material available at FigShare: https://doi.org/10.25386/genetics.9742577.

## Results

tRNAs that mistranslate at different codons and with a range of efficiencies will enable broad applications of mistranslation. We previously demonstrated that *SUP17*, the gene encoding tRNA^Ser^, is lethal when modified with a UGG anticodon and transformed into a wild-type yeast strain (Berg *et al.* 2017). Second-site mutations (*e.g.*, G9A and G26A) that cripple the tRNA allow the mistranslation to be tolerated. By showing that the tRNAs suppress the stress sensitivity of a *tti2**-L187P* mutation, we demonstrated that they mistranslate proline codons, a result confirmed by mass spectrometry. In addition, these mistranslating tRNA^Ser^_UGG_ variants induce a cellular heat-shock response.

**Figure 1:**
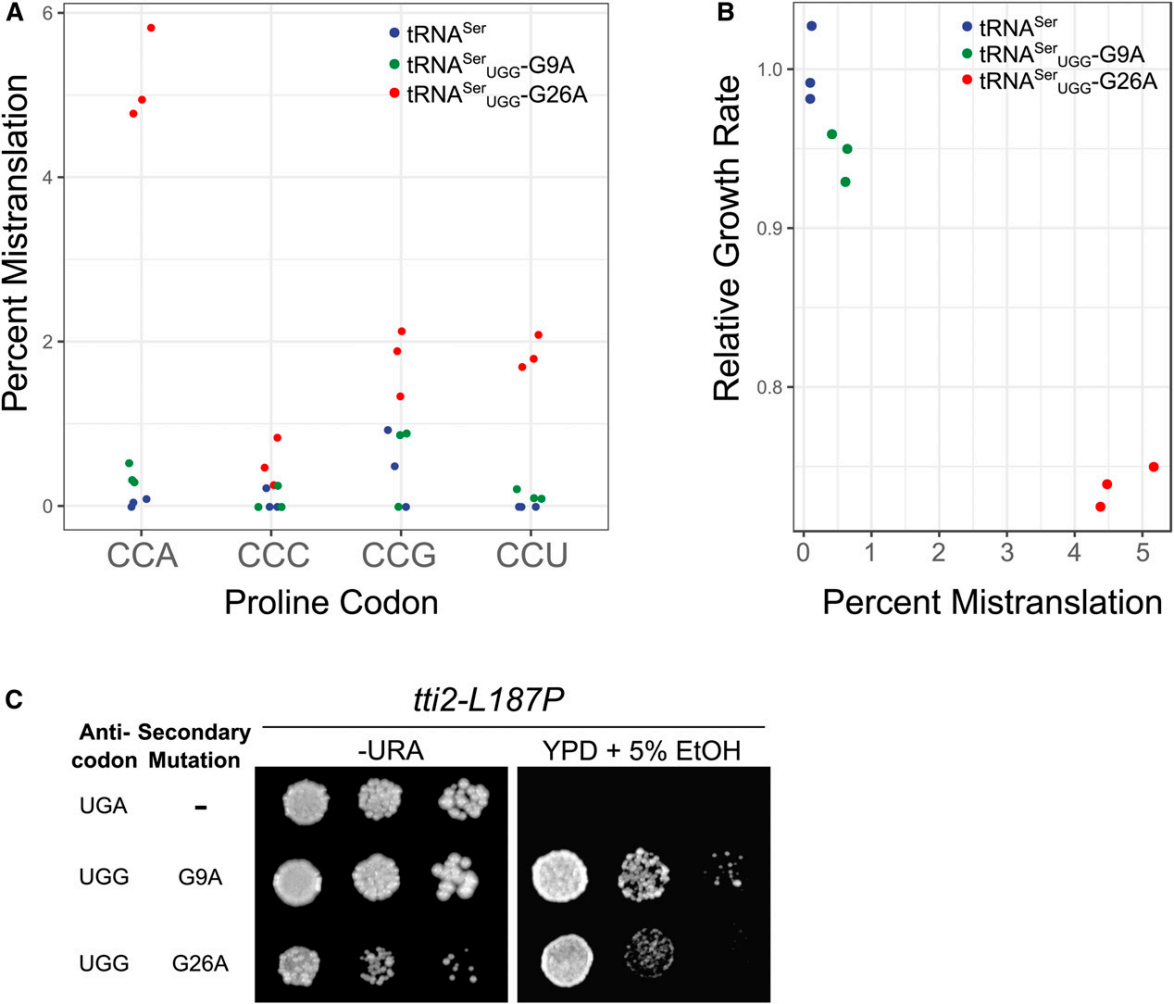
tRNA^Ser^ with a proline UGG anticodon and various secondary mutations allow nonlethal levels of mistranslation. (A) Mass spectrometry analysis of the cellular proteome was performed on wild-type strain (BY4742) containing either wild-type tRNA^Ser^, tRNA^Ser^_UGG_-G9A or tRNA^Ser^_UGG_-G26A. Mistranslation of serine at proline codons was quantified at all four proline codons. (B) Growth rates for each strain in A were determined from growth curves of the strains diluted to an OD_600_ of ∼0.1 in media lacking uracil and grown for 24 hr. Doubling time was calculated with the R package “growthcurver” (Sprouffske and Wagner 2016), normalized to the strain containing the wild-type tRNA and plotted against the percent mistranslation at all proline codons determined through whole proteome mass spectrometry. (C) Yeast strains containing *tti2**-L187P* (CY7020) and either wild-type tRNA^Ser^, tRNA^Ser^_UGG_-G9A or tRNA^Ser^_UGG_-G26A were grown to saturation in media lacking uracil and spotted in 10-fold serial dilutions on media lacking uracil or YPD containing 5% ethanol.

To determine the range of mistranslation induced by tRNA^Ser^_UGG_-G9A and -G26A, we analyzed the cellular proteome by mass spectrometry. The frequency of mistranslation detected at CCA codons in a strain containing a wild-type tRNA^Ser^ was 0.04% (Figure 1A, number of peptides identified can be found in Table S3). The extent of mistranslation for tRNA^Ser^_UGG_-G9A and -G26A was 0.4 and 5.2%, respectively, at the CCA codon. We note that the difference between the G9A variant and the wild-type tRNA was statistically significant (Welch’s *t*-test; *P* < 0.05), although the values do approach the estimated FDR. The majority of mistranslated codons were the cognate CCA codon, but we also observed mistranslation by tRNA^Ser^_UGG_ at the wobble codon CCG and the CCU codon. Decoding of CCU may be due to modification of tRNA^Ser^_UGG_, since tRNA^Pro^_UGG_ in *Salmonella enterica* decodes this codon after modification of U34 to 5-oxyacetic acid (Näsvall *et al.* 2004). The difference in extent of mistranslation by tRNA^Ser^_UGG_-G9A and -G26A was consistent with the lesser effect of the G9A variant on cell growth (Figure 1B). We also observed previously that while both mistranslating variants induce a heat-shock response, G26A induces a greater heat-shock response relative to G9A (Berg *et al.* 2017). Although suppression of *tti2**-L187P* is a sensitive method to detect mistranslation, it is not a quantitative measure of mistranslation (Figure 1C), likely because growth of the *tti2**-L187P* strain under conditions of stress is a balance of both suppression by the tRNA and the toxicity caused by mistranslation.

### Acceptor stem and discriminator mutations can modulate tRNA^Ser^ function

Our goal was to obtain a set of tRNA variants that mistranslate at a broader range of frequencies. Regulating the aminoacylation of a tRNA provides a possible method to modulate functionality (Giegé *et al.* 1998). Based on studies in *E. coli* and *Thermus thermophilus*, the identity elements for tRNA^Ser^ fall within the discriminator base, first 4 bp of the acceptor stem, 1 bp in the D-arm, and unique extended variable arm (Normanly *et al.* 1986, 1992, Himeno *et al.* 1990, 1997; Sampson and Saks 1993; Asahara *et al.* 1994; Biou *et al.* 1994; Saks and Sampson 1996). We found mutations in the variable arm that either decreased the length of the arm, inverted the base pairs, or removed the arm entirely resulted in completely loss of tRNA function and no mistranslation as measured by suppression of *tti2**-L187P* and heat-shock induction (Figure S1). The same was true when we inverted the C11:G24 base pair within the D-arm (Figure S2).

**Figure 2:**
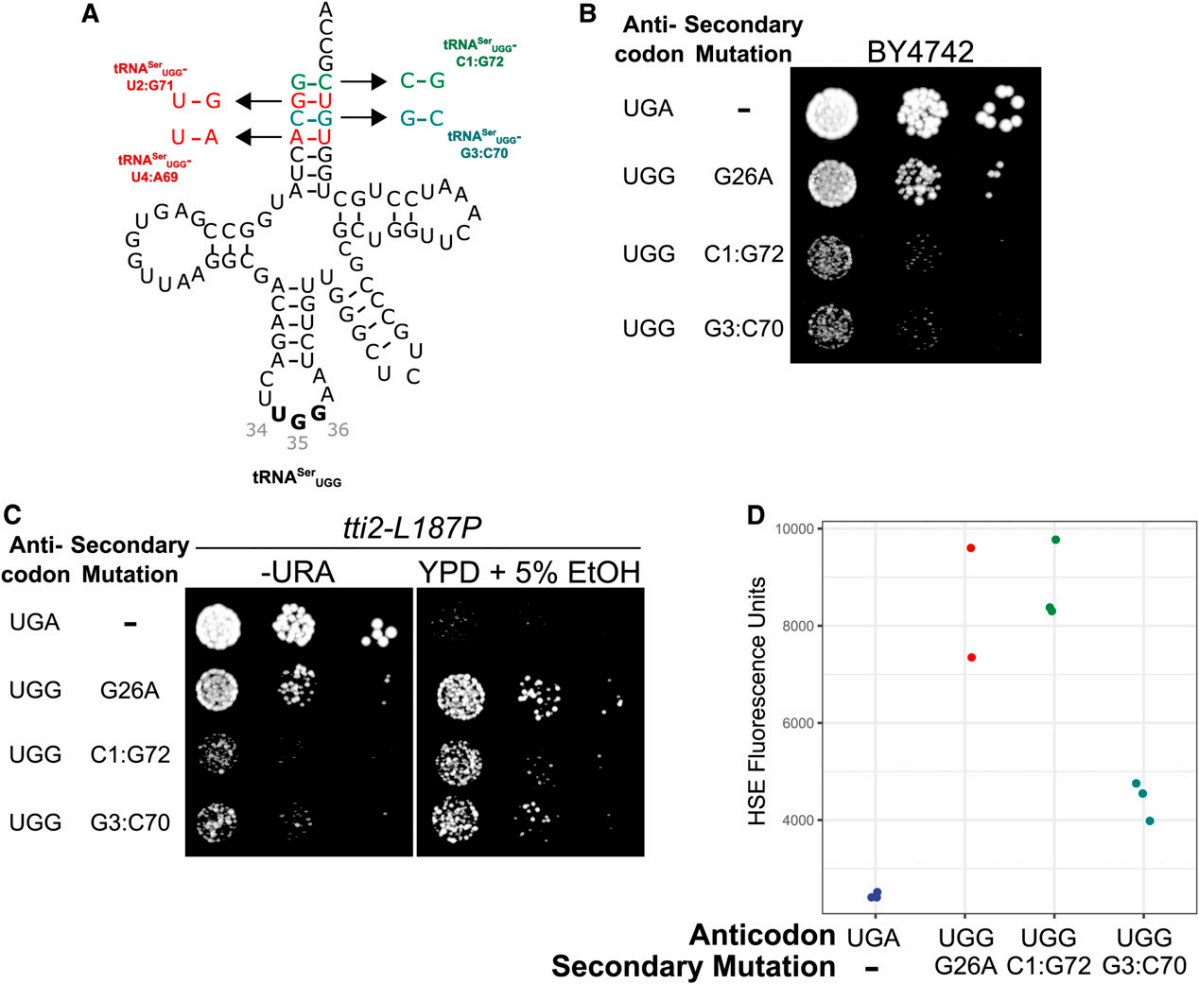
Mutation of G1:C70 and C3:G70 reduces tRNA^Ser^_UGG_ function allowing a nonlethal level of mistranslation. (A) Structures of four tRNA^Ser^_UGG_ alleles created by flipping base pairs at the first four positions in the acceptor stem. (B) Wild-type strain (BY4742) expressing wild-type tRNA^Ser^_UGA_, tRNA^Ser^_UGG_-G26A or the viable tRNA^Ser^_UGG_-C1:G72 or -G3:C70 were grown to saturation in media lacking uracil and spotted in 10-fold serial dilutions on the same media. (C) Strains containing *tti2**-L187P* (CY7020) and one of the tRNAs described in B were grown to saturation in media lacking uracil and spotted in 10-fold serial dilutions on media lacking uracil or complete media containing 5% ethanol. (D) Wild-type strain BY4742 containing one of the tRNAs described in B and a fluorescence heat shock reporter were grown to saturation in media lacking uracil and histidine. Cells were diluted 1:20 in the same media and grown for 6 hr. Cell densities were normalized and fluorescence measured. Each point represents one biological replicate.

We then investigated if bases within the acceptor stem could be mutated to modulate tRNA^Ser^ function and allow mistranslation. Normanly *et al.* (1992) converted a leucine accepting tRNA into a serine accepting tRNA in *E. coli* by mutating positions 2, 3, 70, 71, and 72 within the tRNA^Leu^ acceptor stem to C72, G2:C71 and A3:U70, all conserved within tRNA^Ser^, suggesting that these bases are identity elements for serylation. In addition, Saks and Sampson (1996) have shown that the charging of minihelices by SerRS requires the first 5 bp in the acceptor stem. Therefore, we constructed four alleles each inverting 1 bp in the acceptor stem (Figure 2A). Transformants were not obtained for base inversions at positions 2:71 and 4:69 (Figure S3), suggesting that these variants are highly functional and mistranslate at lethal levels. The variants with base inversions at positions 1:72 and 3:70 could be transformed. Both were partially toxic as measured by reduced growth (Figure 2B), suppressed *tti2**-L187P* (Figure 2C) and induced a heat-shock response (Figure 2D). Together these results suggest that the C1:G72 and G3:C70 base-pair inversions result in a partial loss of tRNA function. The C1:G72 and G3:C70 mutations can thus be used to dampen the function of mistranslating tRNA^Ser^ variants. Interestingly, the G3:C70 variant was more toxic than the G26A variant but the G26A variant induced a greater heat-shock response.

**Figure 3:**
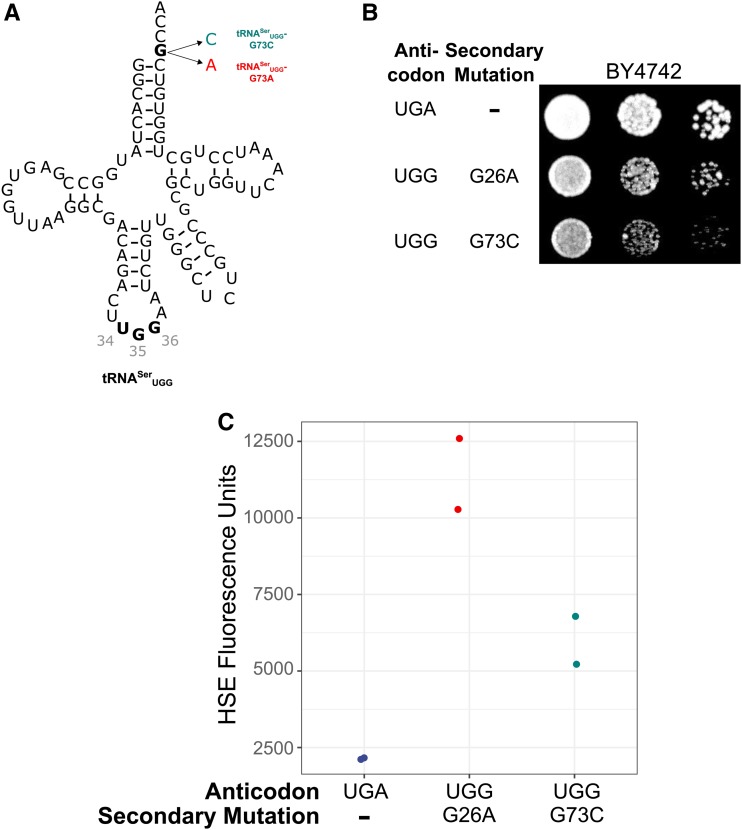
Mutations to the discriminator base of tRNA^Ser^_UGG_ allow mistranslation. (A) Structure of tRNA^Ser^_UGG_-G73C and -G73A. (B) Wild-type strain (BY4742) containing either wild-type tRNA^Ser^_UGA_, tRNA^Ser^_UGG_-G26A or tRNA^Ser^_UGG_-G73C were grown to saturation in media lacking uracil and spotted in 10-fold serial dilutions on media lacking uracil. (C) Wild-type strains containing one of the tRNAs described in B and a fluorescence heat shock reporter were grown in media lacking uracil and histidine. Cells were diluted 1:20 in the same media and grown for 6 hr. Cell densities were normalized and fluorescence measured. Each point represents one biological replicate.

Next, we mutated the discriminator base (position 73), which plays an important role in aminoacylation of many tRNAs by making contacts with the aaRS (Hou 1997; Fukunaga and Yokoyama 2005). All tRNA^Ser^ isoacceptors have G as the discriminator base. We engineered tRNA^Ser^_UGG_-encoding alleles with the discriminator base converted to A or C (Figure 3A) and attempted to transform these alleles on centromeric plasmids into BY4742 (Figure S4). No transformants were obtained for the 73A variant, suggesting that it is highly functional and mistranslates at a lethal level. Transformants expressing the 73C variant were obtained. Consistent with mistranslation by this variant, the transformants grew at a reduced rate relative to the control wild-type tRNA^Ser^ (Figure 3B) and induced a 2.7-fold heat-shock response relative to the wild type (Figure 3C). We again note that similar to the G3:C70 variant, the extent of the heat-shock response did not correlate well with the effect of the G73C variant on growth. We were unable to transform the tRNA^Ser^_UGG_-G73C containing plasmid into the *tti2**-L187P* strain, likely reflecting the combined toxicity of mistranslation with the *tti2* mutation.

In *E. coli* and *Saccharomyces cerevisiae*, the discriminator base for tRNA^Ser^ and tRNA^Leu^ is G and A, respectively. Normanly *et al.* (1992) found that changing the discriminator base of a leucine tRNA from A to the G allowed *E. coli* tRNA^Leu^ to be charged with serine. To test this for *S. cerevisiae* tRNA^Ser^, we constructed tRNA^Ser^ alleles with G73C and G73A mutations in the context of the wild-type UGA serine anticodon. If the tRNA^Ser^_UGA_ variants were mischarged, they should be partially toxic in a wild-type strain and induce a heat-shock response. We did not observe toxicity in growth assays nor did these tRNAs induce a heat-shock response (Figure S5), suggesting that the discriminator base variants are not mischarged. These discriminator base variants are only toxic in the context of a mistranslating tRNA^Ser^ with a noncognate anticodon.

### tRNA^Ser^_UGG_ variants randomly selected to mistranslate at different levels

**Figure 4:**
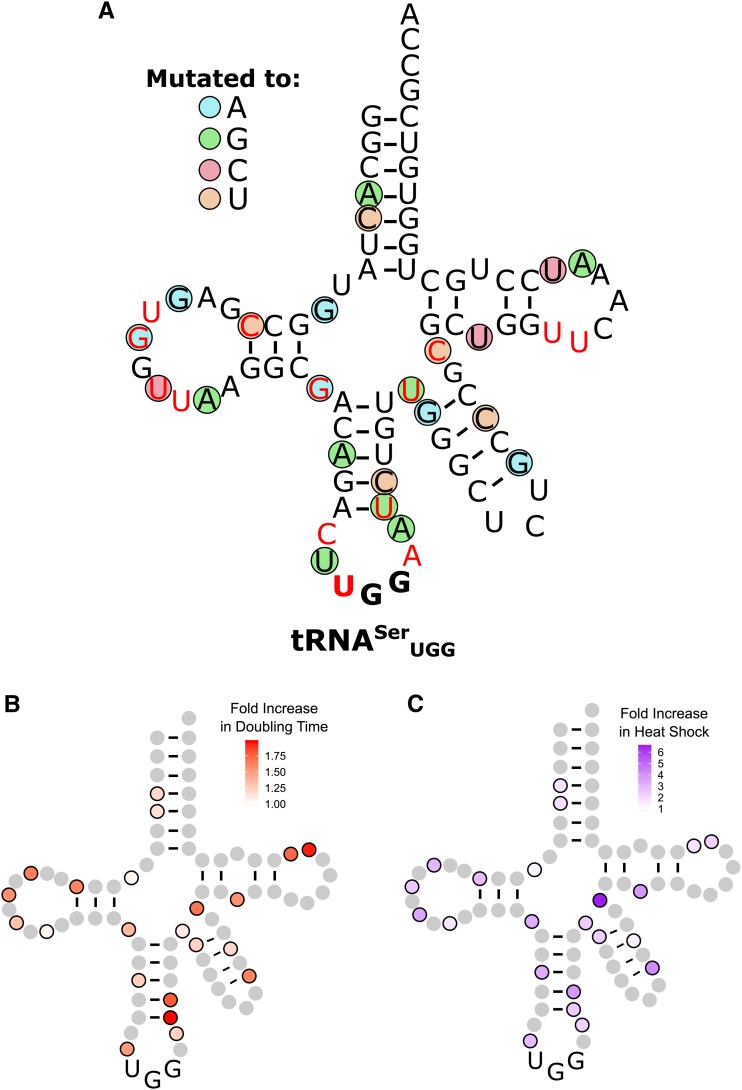
Randomly selected second-site mutations that dampen mistranslation by tRNA^Ser^_UGG_. (A) Base changes are indicated by the colored circles, where blue indicates a mutation to adenine, green to guanine, red to cytosine, and orange to uracil. Bases colored in red are sites of modification in tRNA^Ser^ (Machnicka *et al.* 2013). (B) Heat map of the growth rates of strains containing mutations in tRNA^Ser^_UGG_ that mistranslate at a level that supports viability. BY4742 containing tRNA^Ser^_UGG_ variants were grown to saturation in media lacking uracil, diluted to an OD_600_ of ∼0.1 in the same media and grown for 24 hr at 30°. OD_600_ was measured every 15 min. Doubling time was calculated with the R package “growthcurver” (Sprouffske and Wagner 2016) and normalized to a strain containing a wild-type tRNA^Ser^. (C) Heat map of heat shock induced by tRNA^Ser^_UGG_ derivatives. BY4742 containing different tRNA^Ser^_UGG_ variants and a fluorescence heat shock reporter were grown to saturation in media lacking uracil in biological triplicate. Cells were diluted 1:20 in the same media and grown for 6 hr. Cell densities were normalized and fluorescence measured.

The targeted mutations in tRNA^Ser^_UGG_ that mistranslated resulted in poor viability. To identify additional bases that, when mutated, lower tRNA^Ser^_UGG_ mistranslation to a variety of levels in a less biased manner, we mutagenized the *sup17**(UGG)*-containing plasmid with UV light and randomly selected mutants that suppress *tti2**-L187P*. Plasmids that allowed growth of the *tti2**-L187P* strain on media containing 5% ethanol were isolated, transformed back into the *tti2**-L187P* strain to verify activity, and sequenced (suppression of *tti2**-L187P* by the randomly selected variants is shown in Figure S6). We mapped the mutations onto the tRNA^Ser^_UGG_ secondary structure (Figure 4A). We isolated 22 different mutations that dampen tRNA^Ser^_UGG_ function to allow nonlethal levels of mistranslation, including mutations at G9, A20b, G26, and C40 that we had identified previously (Berg *et al.* 2017). Twelve mutations occur in single-stranded regions; 10 occur in stem structures. Of those occurring in stem structures, six change a Watson–Crick base pair to a G:U pair, including one in the variable arm. The three mutations that abolish base-pairing occur either at the beginning or end of a stem resulting in a shortened stem structure. tRNA^Ser^_UGA_ contains 14 modified bases (Machnicka *et al.* 2013; colored red in Figure 4A). Seven of the mutations that dampen tRNA^Ser^_UGG_ function occur at one of these modification sites.

To estimate the amount of mistranslation by the randomly selected derivatives, we examined their effect on growth in a wild-type strain (Figure 4B and Table 1) and their induction of a heat-shock response (Figure 4C and Table 1). The least toxic tRNA^Ser^_UGG_ variants, containing a C5U, G9A, A20bG, or U44G mutation, had a doubling time similar to the control strain that contained wild-type tRNA^Ser^_UGA_, and induced a 1.9- to 2.4-fold heat-shock response. The most toxic variants with U39G or U60C mutations increased doubling time by 2.0 and 1.7-fold, respectively, and induced a 2.4 and 1.7-fold heat-shock response.

**Table 1:** Mutations to tRNA^Ser^_UGG_ allowing nontoxic levels of mistranslation

Mutation*^a^*	Doubling time (min)	Heat-shock induction (fold change)
tRNA^Ser^_UGA_	65.5	1.0
A20bG	67.4	1.6
G9A	68.3	1.2
U44G	71.6	2.4
C5U	75.5	1.9
Ce21U*^b^*	77.7	1.4
A4G	77.9	1.7
G45A	79.4	2.3
A38G	80.4	2.2
A29G	81.6	3.3
U19C	85.3	3.4
A59G	85.4	2.7
G26A	88.9	3.2
U33G	98.0	2.8
U51C	101	3.8
G17A	102	2.5
C12U	105	2.8
Ge23A*^b^*	105	4.2
G15A	107	2.9
C48U	109	6.5
U60C	113	1.7
C40U	116	3.8
U39G	129	2.4

a With the exception of the wild-type tRNA^Ser^_UGA_, all tRNAs have the UGG anticodon.

b Refers to the extended variable arm, as numbered by Sprinzl *et al.* (1998).

**Figure 5:**
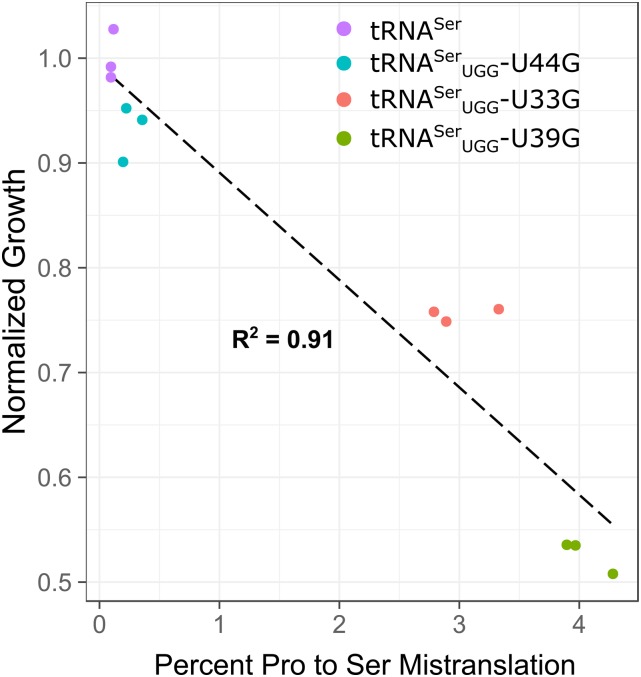
Mistranslation frequency correlates with the effect of each mistranslating tRNA on growth. Whole proteome mass spectrometry was performed on wild-type strain BY4742 containing either a wild-type tRNA^Ser^ or mistranslating variants tRNA^Ser^_UGG_-U44G, U33G, or U39G. Doubling time in minutes for each strain was determined from growth curves of each strain in media lacking uracil and normalized to the strain containing a wild-type tRNA^Ser^.

To better estimate mistranslation frequencies, we analyzed protein lysates by mass spectrometry for tRNA^Ser^_UGG_ variants U44G, U33G, and U39G, which have ∼1-, ∼1.5-, and ∼two-fold increases in doubling time, respectively. We predicted that these variants would mistranslate at different frequencies correlating to their effect on growth. In agreement with this, the U44G variant mistranslated at 0.3%, the U33G variant mistranslated at 3.0% and the U39G variant mistranslated at 4.0% (Figure 5).

**Figure 6:**
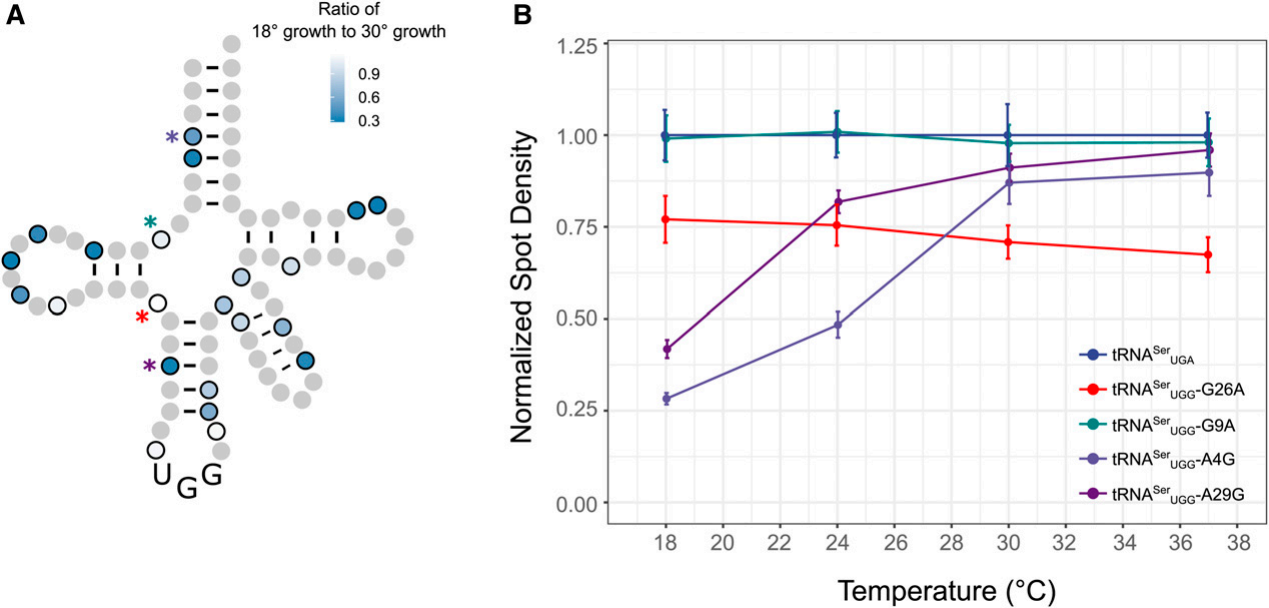
Growth of tRNA^Ser^_UGG_ variants at different temperatures. (A) Wild-type strains containing a mistranslating tRNA^Ser^_UGG_ variant were grown to saturation in media lacking uracil. Optical density was normalized and cultures were diluted 1:100, spotted on media lacking uracil, and grown at either 18° for 4 days or 30° for 2 days. Pixel intensity was measured for each spot. Each value is the average of three biological replicates. (B) Five representative temperature curves from the data presented in A. Strains were grown at 18° for 4 days, 24° for 3 days, or 30° or 37° for 2 days. Each point is the average of three biological replicates and error bars indicate 1 SD.

**Figure 7:**
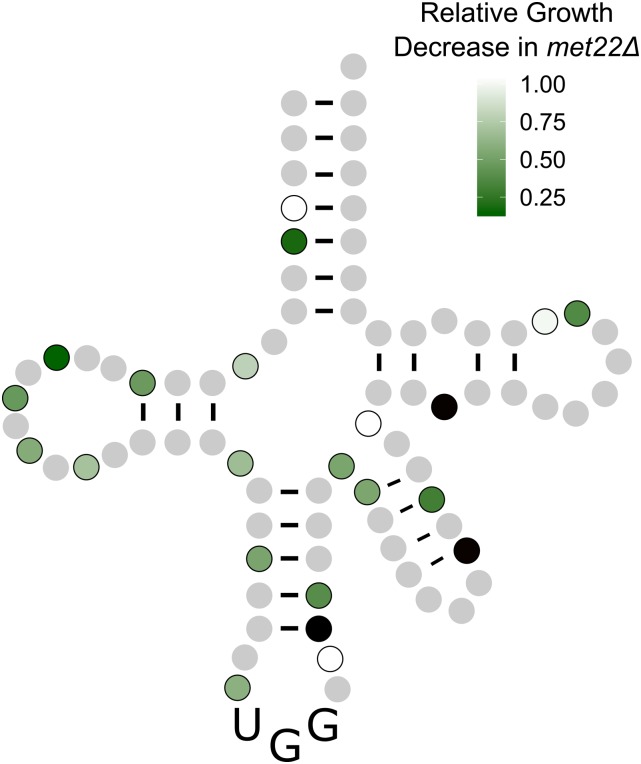
tRNA^Ser^_UGG_ variants that are targets of the RTD pathway. Wild-type *MET22* or *met22*Δ strains containing a mistranslating tRNA^Ser^_UGG_ variant were grown to saturation in media lacking uracil, diluted, and spread for single colonies on media lacking uracil. Colony size was measured using ImageJ after 2 days of growth at 30° and the ratio of *MET22* to *met22*Δ colony size determined for each mistranslating variant. Variants whose growth was not statistically different between the two strains, as determined by a Welch’s *t*-test, are colored white. Three variants where we could not obtain colonies in the *met22*Δ strain are colored black.

To evaluate whether the secondary mutations in the randomly selected variants reduced the stability of the tRNAs, we compared their toxicity at 18° and 30° (Figure 6A with representative temperature curves shown in Figure 6B). Seventeen of the 22 mutations became more toxic at 18°, suggesting that these mutations affect the stability or folding of the tRNA. Neither the G9A nor G26A variants were more toxic at lower temperatures (Figure 6B), suggesting that temperature induced toxicity is not due to changes in translation decoding at different temperatures. Furthermore, this result suggests that the extent of mistranslation can be regulated by growth temperature. To further evaluate if the secondary mutations affect the stability of the tRNA, we determined the toxicity of the mistranslating tRNAs in a *met22*Δ strain where the RTD pathway is inhibited (Dichtl *et al.* 1997; Chernyakov *et al.* 2008). If a mutation reduces tRNA function by increasing the turnover rate of the tRNA, the tRNA should be more toxic in the *met22*Δ strain. Eighteen of the 22 randomly selected tRNA^Ser^_UGG_ variants were more toxic in the *met22*Δ strain than the wild-type strain (Figure 7 and Figure S7). Of the four variants not affected by the RTD pathway, one changed an A:U base pair in the acceptor stem to a G:U and three had mutations in loop regions. We note that of the 22 variants, all but the A38G variant showed either temperature-dependent toxicity or increased toxicity when the RTD pathway was inhibited, or both.

### Mistranslating serine at other codons

**Figure 8:**
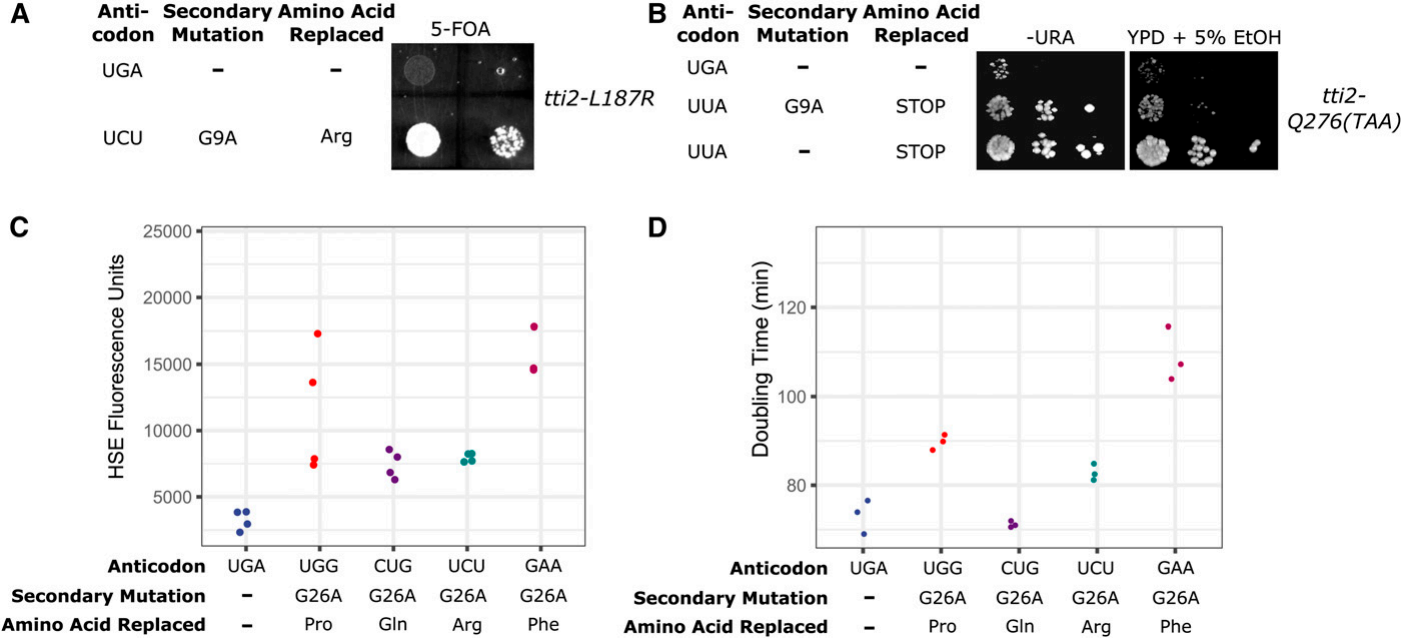
tRNA^Ser^ variants mistranslate at nonserine codons. (A) *tti2**-L187R* and either a wild-type tRNA^Ser^_UGA_ or tRNA^Ser^_UCU_-G9A that mistranslates serine at arginine codons were transformed into the *tti2* disruption strain CY6963 with *TTI2* on a *URA3* plasmid. Strains were grown in media lacking histidine and leucine and plated in 10-fold serial dilutions on 5-fluoroorotic acid containing medium to select against colonies containing wild-type *TTI2*. (B) Wild-type tRNA^Ser^_UGA_, an ochre suppressor serine tRNA (tRNA^Ser^_UUA_) or tRNA^Ser^_UUA_-G9A were transformed into a *tti2* disruption strain containing *tti2**-Q276(TAA)*. Strains were spotted in 10-fold serial dilutions on media lacking uracil or on YPD containing 5% ethanol. (C) Mistranslating tRNAs induce a heat-shock response. Wild-type strain (BY4742) containing a fluorescence heat shock reporter was transformed with wild-type tRNA^Ser^_UGA_, tRNA^Ser^_UGG_-G26A, tRNA^Ser^_CUG_-G26A, tRNA^Ser^_UCU_-G26A, or tRNA^Ser^_GAA_-G26A. Strains were grown to saturation in selective minimal medium, diluted 1:20 in the same media, and grown for 6 hr. Cell densities were normalized and fluorescence measured. Each point represents one biological replicate. (D) Strains containing either wild-type tRNA^Ser^ or mistranslating serine tRNAs from C were grown to saturation in media lacking uracil, diluted to an OD_600_ of ∼0.1 in the same media and grown for 24 hr. OD_600_ was measured every 15 min. Doubling time was calculated with the R package “growthcurver” (Sprouffske and Wagner 2016). Each point represents one biological replicate.

As shown by Zimmerman *et al.* (2018) in yeast and by Geslain *et al.* (2010) in mammalian cells, the ability to change the anticodon of tRNA^Ser^ without affecting serylation makes it ideal to engineer mistranslation at many codons. Interestingly, Zimmerman *et al.* (2018) found that different amino acid substitutions are not equally toxic. To determine if secondary mutations dampen toxicity and allow mistranslation at other codons, we constructed tRNA^Ser^ expressing plasmids with anticodons to decode arginine, ochre, glutamine, and phenylalanine codons. tRNA^Ser^_UCU_ to decode arginine was examined first. No transformants were obtained when we attempted to introduce a centromeric plasmid expressing tRNA^Ser^_UCU_ into a wild-type yeast strain (Figure S8), indicating its toxicity. To decrease mistranslation frequency and allow viable levels of serine for arginine mistranslation, we engineered a plasmid expressing tRNA^Ser^_UCU_ with a G9A secondary mutation, which transformed into cells (Figure S8). To verify mistranslation, we transformed the plasmid expressing tRNA^Ser^_UCU_-G9A into a strain deleted for *TTI2*, but with wild-type *TTI2* on a *URA3* plasmid and *tti2**-L187R* on a *LEU2* centromeric plasmid (CY8607). As shown by plasmid shuffling on 5-fluoroorotic acid medium (Figure 8A), tRNA^Ser^_UCU_-G9A suppresses *tti2**-L187R*, in contrast to the control wild-type tRNA^Ser^_UGA_. Since Tti2 with serine at position 187 supports viability (Berg *et al.* 2017), this result indicates that tRNA^Ser^_UCU_-G9A mistranslates serine for arginine.

Previously, we identified an allele of *tti2* where the glutamine codon at position 276 was mutated to an ochre stop codon (Hoffman *et al.* 2016). *tti2**-Q276TAA* grows slowly on complete medium and is stress sensitive. We predict that tRNA^Ser^ could be engineered to suppress ochre stop codons. As shown in Figure 8B, a wild-type tRNA^Ser^ variant with an ochre anticodon (UUA) is not toxic and suppresses the *tti2**-Q276TAA* slow growth and stress-sensitive phenotype. In this case, the tRNA^Ser^_UUA_ lacking secondary mutations was not lethal, likely because of competition between the tRNA and release factor. The variant with the ochre anticodon and a G9A secondary mutation was less active, suppressing the slow growth of *tti2**-Q276TAA* on complete media and only weakly restored growth on medium containing 5% ethanol.

We generated serine tRNAs with glutamine (CUG) or phenylalanine (GAA) anticodons in the context of a G26A secondary mutation, to prevent potential lethality. Since we do not have a reporter construct to detect mistranslation of glutamine or phenylalanine codons, we used heat-shock response and growth rates as proxies for mistranslation. Heat-shock response was measured in the wild-type BY4742 strain expressing wild-type tRNA^Ser^_UGA_ or G26A variants that decode either glutamine, phenylalanine, proline, or arginine codons. All of the strains expressing a variant tRNA induced a heat-shock response, as compared to the strain containing the wild-type tRNA^Ser^_UGA_ (Figure 8C). The effect of the tRNAs on growth is shown in Figure 8D. The serine tRNA with a glutamine anticodon did not affect growth, whereas the tRNA decoding arginine, proline, or phenylalanine significantly increased doubling time by 10, 20, and 50%, respectively (Welch’s *t*-test; *P* < 0.05), indicating that incorporating serine for different amino acids has different consequences. Our results also demonstrate that serine misincorporation at different codons can be achieved by pairing with an appropriate secondary mutation.

## Discussion

tRNAs that mistranslate the genetic code have utility in a number of molecular and synthetic biology applications for both research and biotechnology. Because of its effects on the proteome, mistranslation can be toxic or even lethal, depending on the level of mistranslation and the amino acid substitution. For mistranslation to be useful in biological applications, it must be modulated to avert toxicity. Here, we identify secondary mutations within the tRNA to allow for a range of mistranslation levels for serine to proline substitution (all variants investigated are listed in Table S5). These same derivatives are applicable for substitutions at other codons.

Uses of mistranslation include expanding the genetic code to incorporate noncanonical amino acids, generating statistical proteins that have expanded activity or substrate specificity or inducing proteome wide translational errors to study the effects of amino acid substitutions on cellular functions. The range of efficiencies allows selection of the optimal variant for the application. For example, selecting the optimal tRNA^Ser^ variant could allow incorporation of noncanonical amino acids or even selenocysteine in yeast, without resulting in a loss of fitness, which can lead to poor protein yields. Tuning of the mistranslation will also facilitate optimal mistranslation levels when using multiple tRNAs that substitute at different codons. The temperature-sensitive nature of the tRNAs variants we identified allows regulated mistranslation and applications such as the control of protein expression when used to suppress stop codons. For example, a stop codon could be incorporated early in the protein coding sequence of interest. In the presence of a corresponding temperature-sensitive tRNA^Ser^ derivative, at low temperatures the stop codon would be read through and the protein expressed. The conditional mistranslation also allows mistranslation at levels above the lethal threshold.

### Generating tRNAs that mistranslate at different levels

We found that an unbiased genetic selection was the most efficient approach to identify tRNA^Ser^ bases with nonlethal levels of mistranslation. Bases altered in the targeted strategy were often essential and many of the variants did not mistranslate. By selecting for randomly induced mutations of tRNA^Ser^_UGG_ that suppressed *tti2**-L187P* and were therefore mistranslating at a nonlethal level, we identified 22 single nucleotide mutations that induce mistranslation, including mutations on G9, A20b, G26, and C40 that we had identified previously (Berg *et al.* 2017). Most of these changes occur in single-stranded regions (12 variants) or create a G:U pair (six variants), and thus they likely maintain the overall tRNA structure. The mutations that abolish base pairing occur at the beginning or end of the stem resulting in its shortening. Despite this, we conclude that most of the secondary mutations have a role in tRNA stability and/or turnover, since 21 of the variants were more susceptible to the RTD pathway (18 variants) and/or showed enhanced toxicity at low temperature (19 variants). Included in the latter were base changes that altered base pairing (A4G, C5U, C12U, A29G, U39G, C40U, G45A, Ge23A, and Ce21U). Many of these create a G:U base pair, thus supporting the idea that although thermodynamically similar to a Watson–Crick pair, the G:U pair has different structural and chemical properties that alter tRNA structure (Varani and McClain 2000).

We considered that the enhanced toxicity of the mistranslating variants at lower temperatures may be due to a temperature-dependent effect of mistranslation on cells, rather than being an effect of tRNA stability. However, neither the G9A nor G26A variant (shown in the representative temperature curves in Figure 7B), with mistranslation frequencies of <1% and ∼5%, were more toxic at lower temperatures. Their lack of increased toxicity suggests that the temperature-induced toxicity seen for other variants is a result of tRNA stability, rather than a temperature-dependent effect of mistranslation on the proteome.

The concentration of a tRNA in the cell depends upon both its synthesis and degradation. We note that six variants had base changes in the A box or B box region, required for transcription by RNA polymerase III (Hamada *et al.* 2001). The EufindtRNA algorithm, a tool that predicts likelihood of tRNA expression based upon a consensus sequence (Pavesi *et al.* 1994), predicts that all the variants will be expressed and only the G17A secondary mutation substantially decreases the Eufind score (Table S4). As these variants are all temperature sensitive, we suggest the base changes have a more pronounced effect on stability than synthesis.

Nucleotide changes that affect tRNA stability yet enable partial activity *in vivo* are difficult to predict. Therefore, using a genetic selection as we have done here or a screen for alleles that induce a partial heat-shock response appears to be the most efficient way to identify mistranslating tRNA variants.

### Features of the sequence-function relationships of S. cerevisiae tRNA^Ser^

With the exception of mitochondrial tRNA^Ser^ in metazoans (Helm *et al.* 2000), the long variable arm is conserved in all tRNA^Ser^ molecules and is a key element in recognition by SerRS (Normanly *et al.* 1986, 1992; Sampson and Saks 1993; Asahara *et al.* 1994; Biou *et al.* 1994; Himeno *et al.* 1997; Bilokapic *et al.* 2006). Our results indicate the importance of both the length and sequence orientation of the variable arm for function of tRNA^Ser^ in *S. cerevisiae*. As little as a single base-pair deletion impaired function below a detectable level in the sensitive *tti2**-L187P* suppression assay. Similarly, reversing the orientation of the G:C base pairs in the variable arm eliminated function. Our results also confirm the essential nature of the D-arm C11:G24 base pair of tRNA^Ser^_UGG_, previously identified for its role in serylation in *E. coli* (Normanly *et al.* 1986, 1992; Asahara *et al.* 1994).

SerRS is a class II synthetase that contains a seven-sheet, antiparallel β-fold (Artymiuk *et al.* 1994). In this fold, motif 2 interacts with the acceptor stem (Burke *et al.* 2000) and models of the interaction position motif 2 close to base pairs 3:70 of the acceptor stem (Eichert *et al.* 2011; Berg *et al.* 2018). Our results indicate that base pairs 1:72 and 3:70 play partial roles in tRNA^Ser^ function in *S. cerevisiae*, whereas base pairs 2:71 and 4:69 have lesser roles. This is consistent with a previous study, where we found that cross-species differences in the function of human and yeast tRNA^Ser^ functions are the result of differences at the 3:70 position (Berg *et al.* 2018).

Modifications play many roles in tRNA function, from facilitating accurate decoding to maintaining the correct tRNA structure (Lorenz *et al.* 2017). Fourteen bases in tRNA^Ser^ are modified (Machnicka *et al.* 2013). In our random selection for reduced function, we identified mutations at seven of these modified bases. Six were more toxic at lower temperatures and were turned over by the RTD pathway consistent with the modifications enhancing folding and protecting the tRNA from degradation at higher temperatures. Also consistent with this idea, thermophiles have more abundant and diverse tRNA modifications, including thiolations and methylations, which rigidify the tRNA structure (Watanabe *et al.* 1976; Kawai *et al.* 1992; Kowalak *et al.* 1994; McCloskey *et al.* 2001).

### Cellular effects of mistranslation

The effect of the tRNA^Ser^_UGG_ variants on doubling time varied from almost no change in growth to a twofold increase in doubling time. The change in growth correlated well with the frequencies of mistranslation as estimated by mass spectrometry for the five mistranslating tRNAs that were tested. Based on this correlation, we predict mistranslation rates as high as 8% could be achieved with a 90% decrease in fitness. This is consistent with other measures for the maximum tolerable amount of mistranslation. For example, Ruan *et al.* (2008) found *E. coli* can tolerate up to 10% mismade protein and Mohler *et al.* (2017) found yeast can tolerate 8% tyrosine incorporation at phenylalanine codons. We also note that based on the correlation we observed, growth rate provides a better proxy for mistranslation than level of heat-shock response.

We compared the growth characteristics of strains mistranslating at proline, arginine, glutamine, and phenylalanine codons. Of these, misincorporation at phenylalanine codons resulted in the most pronounced decrease in growth rate. A similar result was obtained by Zimmerman *et al.* (2018), who found that tRNA^Ser^ with phenylalanine anticodons were depleted from pools of cells containing tRNA^Ser^ with randomized anticodons. Zimmerman *et al.* (2018) discuss possible reasons for differences in toxicity noting that level of misincorporation does not correlate well with toxicity when comparing different amino acid substitutions. Reasons include whether the amino acid substitution is conservative or nonconservative, features of codon usage, and the ratio of the mistranslating tRNA to the native isodecoder. Zimmerman *et al.* (2018) investigated the effect of competition between a mistranslating tRNA and native cognate tRNA on toxicity, finding that overexpressing a wild-type cognate tRNA reduced the toxicity of mistranslation. The mistranslating serine derivatives generated here that mistranslate at phenylalanine, proline, arginine, or glutamine codons all compete with approximately the same number of wild-type cognate tRNAs (10 for GAA phenylalanine, 10 for UGG proline, 11 for UCU arginine, and 10 for GUC glutamine). We believe that the specific chemistry of the substitution explains the varying toxicities of mistranslation at different codons, although we recognize that the analysis is complicated by difficulties quantitating all of the parameters in cells; for example, the extent and efficiency of wobble.
